# Ensuring the Voice of the Very Severely Affected Myalgic Encephalomyelitis/Chronic Fatigue Syndrome Patient Is Heard in Research—A Research Model

**DOI:** 10.3390/healthcare10071278

**Published:** 2022-07-10

**Authors:** Helen Baxter

**Affiliations:** 25% ME Group, Troon KA10 6HT, UK; hbaxter@25megroup.org

**Keywords:** Myalgic Encephalomyelitis/Chronic Fatigue Syndrome (ME/CFS), severely affected, research, maximising participation, reasonable adjustments, direct outreach, telephone and text support

## Abstract

Most of the research about Myalgic Encephalomyelitis/Chronic Fatigue Syndrome (ME/CFS) has focused on ambulant patients who are able to attend clinics. It is estimated that 25% of people with ME/CFS are severely, or very severely, affected and are housebound or bedbound; some require tube feeding. Due to the severity of their illness, these patients have largely been excluded from research and are often described as ‘hard to reach.’ A questionnaire was devised to gather data about their experiences of accessing tube feeding. By making the necessary reasonable adjustments, such as direct outreach and the option to complete the questionnaire by telephone or texting, very severely affected patients were enabled to participate and provided invaluable contributions. This study aimed to act as a model for future researchers.

## 1. Introduction

Myalgic Encephalomyelitis/Chronic Fatigue Syndrome (ME/CFS) has a prevalence of 0.2–0.4% in the population [1], equating to approximately 250,000 people in the UK. An estimated 25% have severe, or very severe, ME/CFS, and are housebound or bedbound [2]; some of the most severely affected will require tube feeding. ME/CFS has historically received insufficient funding for research, particularly when compared to other conditions, such as multiple sclerosis [3]. Research into severe ME/CFS is very limited. A special edition of *Healthcare* focused on severe, and very severe, ME/CFS led to the publication of 25 papers [4]. However, none looked at ways to increase participation of severely affected ME/CFS patients in research. This study examined how to engage patients who have been described as the ‘hidden patient population [4]’, who have previously been largely excluded from research, due to the severity of their ME/CFS and are often described as ‘hard to reach’ [5]. Stasheim et al. recorded a poor response rate as well as questionnaires being returned incomplete [6], when trying to engage severely affected patients. Lacerda et al. highlighted the increased time and cost associated with research into severe ME/CFS [7]. The 25% ME Group, a national charity which supports and advocates for people with severe, and very severe, ME/CFS, became aware of the significant clinical delays being experienced by patients requiring enteral or parenteral nutrition, and became concerned about the clinical responses, so wanted to collect data from charity members. A study questionnaire was devised which recognised previously encountered difficulties experienced when researching severe ME/CFS. It considered the issues likely to prove a barrier to completing the document and tried to maximise participation, whilst reducing the risk of health deterioration. The aims of the study presented here were to explore whether, if the necessary support was provided and a modified extension to the usual timescale given, patients with very severe ME/CFS would be enabled to participate in research.

Rather than focussing on data analysis or the precision of data collected, this paper describes the methods of collecting data from patients with severe ME/CFS, and the process of achieving this, and it aims to act as a model for future researchers wanting to undertake research with patients with very severe ME/CFS.

## 2. Materials and Methods

Previous studies have struggled to recruit and retain participants. Difficulty retaining participants was demonstrated in research undertaken by Geraghty et al., which focussed on people with severe ME/CFS. In their study, approximately 1600 people clicked on the survey link, 343 started the survey, but only 124 participants completed all the questions. Of those who initially expressed interest in the study, only 7.75% completed the questionnaire [8]. Similarly, Strasheim et al. posted 483 questionnaires to participants and only 63 were returned ‘in various stages of completion’ [6].

With this in mind, the 25% ME Group devised a questionnaire for members who had experience of being enterally and parenterally fed. The questions were not based on previous research, as no previous research had been found.

The investigator had significant experience dealing with people with severe, and very severe, ME/CFS, which meant she understood the cognitive difficulties faced by people with ME/CFS, and, thus, the survey was designed such that it was clearly stated which questions needed to be completed, depending on the type of artificial nutrition (AN) being received. This avoided energy being wasted looking through questions. Questions were simple and included age, reason for, and duration of, AN, as well as an open-ended section in which participants could provide further information (see Appendix A). This contrasted with the questionnaires chosen by Strasheim et al., namely, the DePaul Fatigue Questionnaire and the Barthel Functional Outcome Measure, both of which are complex and lengthy to complete. Feedback from ME North East clients expressed the “difficulties they had concentrating on the questionnaire and supporting governance paperwork” [6]. This study provided assistance with the paperwork. In Summer 2019, an invitation was placed in the 25% ME Group charity’s newsletter, ‘The Quarterly’, inviting members who met the criteria to complete the questionnaire. It was placed on the fourth page, giving it a greater chance of being seen by members who can often only, at best, look at the newsletter in small amounts. The questionnaire was available via email or post. Direct outreach was used for members who were too ill to read the newsletter and, once aware of the questionnaire, members were keen to participate. Aware that, due to the severity of their ME/CFS, they were not going to be able to complete the questionnaire unaided, the investigator completed it with them by telephone or by text. Appointments were made on the understanding that they might need to be changed by the participant at short notice and would end if the participant felt unwell. The investigator’s experience in talking to people with very severe ME/CFS meant that, when speaking to participants, she spoke in a slow, soft voice and avoided asking them to repeat themselves. She knew participants found it cognitively much less challenging to talk freely about their lived experience, rather than responding to direct questions. By allowing them to do this, the participants did answer the questions posed in the questionnaire and the investigator read their responses back to seek confirmation that they were correct. 

Other reasonable adjustments were put in place to maximise participation, whilst minimising the risk of causing post-exertional malaise (PEM). These included allowing family and homecare workers to assist with completion of the questionnaire. A deadline was not set as the investigator knew the pressures felt, and deteriorations in health encountered, by people with severe ME/CFS when trying to meet deadlines completing official forms.

## 3. Results

Of those who initially expressed interest, five in total, all went on to complete the questionnaire. The results were analysed qualitatively. All participants were female with a mean age of 39 (range from 21 to 55). The duration of their illnesses ranged from 6 years to 32 years, with a mean of 23 years. All participants had a diagnosis of ME/CFS from a National Health Service (NHS) consultant physician and, due to being bedbound and their need for tube feeding, were defined as having very severe ME/CFS, according to NICE Guideline 206 [9]. Recurrent themes occurred, such as clinical inertia, but individuals also provided detailed accounts of their experiences. The open-ended section of the questionnaire, asking for ‘any other relevant information’, received a significant response from all participants. The information provided was used to create a series of case reports [10].

One member and the families of a further two members requested the questionnaire by email. One questionnaire was completed as an online document, the other two were printed and returned as email attachments.

Only one member was well enough to complete the questionnaire independently. One questionnaire was completed by the parents with input from the patient, whilst another questionnaire was solely completed by the family member who cared for the person. Of the two members requiring direct outreach, one questionnaire was completed using a combination of information provided by the person through their home care worker, as they had lost the ability to speak, and texting, whilst the other was completed over the telephone in multiple appointments. Appointments were cancelled due to participants experiencing problems, such as migraine and seizures and, on several occasions, had to be terminated during the conversation due to the participant becoming unwell. 

The length of time required to complete the questionnaire varied: in one case, where it was being completed by a family member, it took three days, while in another it took a year to gather the necessary information, due to the severity of the patient’s illness and the need for significant rest between appointments. 

### Scope and Limitations of Study

It is not known how many charity members are receiving AN and, thus, whether there were an additional number of patients who were not reached by this methodology. Due to the small sample size (n = 5) any conclusions drawn could only be tentative. This is a pilot study which needs to be replicated using a larger sample size. 

## 4. Discussion

Multiple questionnaires [6] and surveys with multiple questions [8] previously led to a high dropout rate with a resultant collection of limited qualitative data of patients’ lived experiences [8]. However, the simplicity of this survey meant that all those who initially expressed interest were able to complete it using the options available.

Other research [6] and responses from patient participation groups [7] have shown a keenness amongst patients to participate in research and that:

“with increased support to the participants, more could be realised”

[6]

This study was able to provide the increased support by collecting the information from the participants in a form most accessible for them. The addition of telephone and text support enabled two of the study’s most severely affected patients to participate. Without this support they would have been unable to do so.

Geraghty et al. [8] identified a need for researchers to have expert knowledge of ME/CFS. In this study, the investigator had previously worked with people with severe, and very severe, ME/CFS and drew on this in the design of the survey, the direct outreach and the completion methods of the survey. Such was the keenness of the participants to provide information that, on occasion, it was necessary for the investigator, when conducting telephone interviews, to say to the participant, “You’re sound tired shall we leave it for today?” Her experience enabled her to listen for sounds of fatigue in the patient’s voice. It is imperative that participating in research does not cause a decline, either short term or long term, in the patients’ health.

Some patients with severe, and very severe, ME/CFS will not have access to the internet. This could be due to an inability to tolerate the sensory stimulus from a screen or to financial constraints. Thus, surveys which are only promoted on online platforms [8] will inevitably exclude some of the most severely affected. The invitation to participate in this survey was printed in a paper copy of the charity newsletter, to be sent to all members. The further step of using direct outreach for members too ill to read the newsletter increased the uptake by two fifths.

Likewise, surveys which require completion on screen may not be possible and the participant may not have anyone to help them. A reduction in social care provision in the UK means home care workers do not have sufficient time to assist patients with the completion of research surveys. Furthermore, the information patients provide may be something they do not wish to share with their homecare worker; thus, the role of the investigator becomes crucial.

This survey was produced as a Word document making it possible for participants who wished to complete it on screen to save it and continue completing it as and when their health allowed. It was noteworthy that the only questionnaire completed on screen was done without direct input from the person with very severe ME/CFS. Future research needs to ensure appropriate software is used to enable participation.

One participant took a year to complete the questionnaire, due to the severity of her illness. This contrasted with the research on severely affected ME/CFS patients undertaken by Geraghty et al. [8], where the survey was only open for a week and was closed as ‘a sufficient number of responses’ had been received. Whilst their research provided quantitative data, it might have compromised on qualitative data. Not imposing a deadline in the study here allowed participants to prioritise their health, but to still complete the survey.

Geraghty et al. [8] recognised the difficulty face to face visits for interviews can pose for severely affected patients and deemed telephone appointments unfeasible, based on feedback from patients. However, this study showed telephone and text appointments, when used flexibly, can be very effective in obtaining research material. When listening to the patient the researcher was able to obtain answers to questions posed in the survey and other relevant information without needing to ask direct questions which, due to cognitive impairment, patients could find difficult. The geographical location of the patient ceased to be relevant. Patients were more likely to cancel an appointment at short notice if it was to be conducted via telephone, thus reducing any impact on their health. Costs incurred by the study were significantly reduced by providing additional support using this method. By providing telephone and text support, where participants needed assistance with completing surveys, not setting deadlines, and avoiding doing home visits, which participants can find fatiguing and which can cause Post Exertional Malaise, this study advanced previously used methods.

However, where physical samples are required, it is imperative domiciliary visits are provided [7,11]. It is necessary to include the additional costs identified in the research budget; for example, printing paper copies of surveys.

According to [4], “Although never formally studied, it is estimated that twenty-five percent of ME/CFS patients are either severely or very severely affected”, and, thus, without quantitative research it is impossible to say how many participants would need such an approach to participate.

## 5. Conclusions

Although patients with severe ME/CFS have often been excluded from previous research, due to being regarded as ‘hard to reach’, this study showed that if people with very severe ME/CFS are made aware that research is being undertaken, and the necessary support is provided, without time constraints, they can make invaluable contributions to research.

Recommendations to ensure high quality research in the form of a checklist for future researchers to follow:

- Utilise organisations, charities and support groups, both locally and nationally, who know the demographics of their patients, or members.
- Contact charities and other organisations to publicise an invitation to take part in research, both online and in print.
- Liaise with charities to find out when they send members printed documents, such as magazines, and place advertisements in these, allowing sufficient time for potential participants to see the advertisement and respond to it.
- When designing a survey, use simple language. Aim for a reading age of nine [12].
- Ensure software is used which has a ‘save’ function to enable participants to complete and return the document, as and when their health permits.
- Offer paper copies of surveys/questionnaires.
- Offer assistance to complete the survey/questionnaire by telephone and, if possible, by text.
- Look to recruit people with knowledge of severe ME/CFS to assist with completing documentation by telephone.
- Speak slowly and softly when talking to people with severe ME/CFS and avoid having them repeat themselves. Read the participants’ responses back to them for confirmation that they are correct.
- Make funders aware of the need for extended deadlines.

## Data Availability

The datum presented in this study is available on request from the author.

## Appendix A

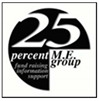

**ENTERAL AND PARENTERAL FEEDING QUESTIONNAIRE**
**Are You:** [Circle all that apply] **NG Fed** go to **Section 1** **NJ Fed** go to **Section 2** **PEG Fed** go to **Section 3** **PEJ Fed** go to **Section 4** **On TPN** go to **Section 5** **Everyone needs to complete Section 6**
**Section 1 NG Fed members only**
Age How long did you have ME before being NG fed? Reasons for NG Feeding How long have you been NG fed? Hospital Consultant Gastroenterologist Who manages your tube on a daily basis i.e., flushing/ aspirating? Who oversees the care of the tube and your nutrition in the community When the tube needs replacing where does this take place? Is there a plan in place for the replacement e.g. elective resiting?
**Section 2 NJ Fed members only**
Age How long did you have ME before being NJ fed? Reasons for NJ Feeding How long have you been NJ fed? Hospital Consultant Gastroenterologist Who manages your tube on a daily basis e.g. flushing? Who oversees the care of the tube and your nutrition in the community? When the tube needs replacing where does this take place? Is there a plan in place for the replacement e.g. elective re-siting? Are you NJ fed due to gastroparesis? Have you been tested for / diagnosed with Mast Cell Activation Disorder? If "YES" to the above, circle as appropriate **Section 3 PEG Fed members only** Age Hospital Consultant Gastroenterologist How long did you have ME for before any form of tube feeding commenced? How long did you have ME before being PEG fed? Were you NG fed before being PEG fed? If so, why was the decision made to site a PEG? Reasons for PEG Feeding How long have you been PEG fed? Who manages your tube on a daily basis e.g. flushing/aspirating? Who oversees the care of the tube and your nutrition in the community? **Section 4 PEJ Fed members only** Age Hospital Consultant Gastroenterologist How long did you have ME for before any form of tube feeding commenced? How long have you been PEJ fed? What other forms of tube feeding were tried prior to PEJ feeding? Why and by whom was it decided you needed a PEJ? Who manages your tube on a daily basis e.g. flushing? Who oversees the care of the tube and your nutrition in the community? Are you PEJ fed due to gastrointestinal failure? Have you been tested for/diagnosed with Mast Cell Activation Disorder? If ‘YES’ to the above, circle as appropriate **Section 5 Total Parenteral Nutrition** Age Hospital Consultant Gastroenterologist How long did you have ME for before any form of tube feeding commenced? How long have you been on TPN? What other forms of tube feeding were tried prior to TPN? How long were they tried for and why were they stopped? Were you allowed to become underweight whilst different types of tube feeding were tried? Who manages your central line on a daily basis? Are you on TPN due to gastrointestinal failure? Have you been tested for / diagnosed with Mast Cell Activation Disorder? If "YES” to the above, circle as appropriate **Section 6 All enteral/parenteral fed members** Have you been assessed by SALT? Were you diagnosed with an unsafe swallow? Were you allowed to become underweight prior to tube feeding commencing? Was your inability to eat ever considered to be anorexia nervosa? Were you ever threatened with sectioning prior to tube feeding commencing? Do you feel your health has improved by being tube fed? Any other relevant information: Thank you for taking the time to complete the questionnaire. June 2019

## References

[1] Nacul L.C., Lacerda E.M., Pheby D., Campion P., Molokhia M., Fayyaz S., Leite J.C., Poland F., Howe A., Drachler M.L. (2011). Prevalence of myalgic encephalomyelitis/chronic fatigue syndrome (ME/CFS) in three regions of England: A repeated cross-sectional study in primary care. BMC Med..

[2] CFS/ME Working Group (2002). Report to the Chief Medical Officer of an Independent Working Group.

[3] Radford G., Chowdhury S. (2016). ME/CFS Research Funding: An Overview of Activity by Major Institutional Funders Included on the Dimensions Database. Action for ME. https:/www.actionforme.org.uk/uploads/pdfs/mecfs-research-funding-report-2016.pdf.

[4] Friedman K., Bateman L., De Meirleir K.L. Special Issue: ME/CFS—The Severely and very Severely Affected. Healthcare.

[5] (2009). Pathways Through Participation. Briefing Paper No. 3—Who Participates? The Actors of Participation?. http://pathwaysthroughparticipation.org.uk/wp-content/uploads/2009/09/Briefing-paper-3-Who-participates1.pdf.

[6] Strassheim V.J., Sunnquist M., Jason L.A., Newton J.L. (2018). Defining the prevalence and symptom burden of those with self-reported severe chronic fatigue syndrome/myalgic encephalomyelitis (CFS/ME): A two-phase community pilot study in the North East of England. BMJ Open.

[7] Lacerda E.M., Kingdon C.C., Bowman E.W., Nacul L. (2018). Using a participatory approach to develop and implement the UK ME/CFS Biobank. Fatigue.

[8] Myalgic Encephalomyelitis (Or Encephalopathy)/Chronic Fatigue Syndrome: Diagnosis and Management. Appendix 2. https://www.nice.org.uk/guidance/ng206/evidence/appendix-2-involving-adults-with-severe-mecfs-symptoms-pdf-333546588759.

[9] NICE Guideline 206 Myalgic Encephalomyelitis (Or Encephalopathy)/Chronic Fatigue Syndrome: Diagnosis and Management. https://www.nice.org.uk/guidance/ng206.

[10] Baxter H., Speight N., Weir W. (2021). Life-Threatening Malnutrition in Very Severe ME/CFS. Healthcare.

[11] Chang C.-J., Hung L.-Y., Kogelnik A.M., Kaufman D., Aiyar R.S., Chu A.M., Wilhelmy J., Li P., Tannenbaum L., Xiao W. (2021). A Comprehensive Examination of Severely Ill ME/CFS Patients. Healthcare.

[12] Flesch Reading Ease Score—Reading and Grade Level Calculator. www.charactercalculator.com/flesch-reading-score.

